# Evaluation of the proximal contact tightness in class II resin composite restorations using different contact forming instruments: a 1-year randomized controlled clinical trial

**DOI:** 10.1186/s12903-023-03462-5

**Published:** 2023-10-07

**Authors:** Karim M. Abbassy, Waleed A. Elmahy, Ahmed A. Holiel

**Affiliations:** Conservative Dentistry Department, Faculty of Dentistry, Alexandria University, Alexandria, Egypt

**Keywords:** Proximal contact, Sectional matrix, Contact forming instrument, Composite restoration, Dentistry, Dental restoration, Permanent

## Abstract

**Background:**

Proper proximal contact in direct composite restorations is crucial for periodontal health. Over a one-year period, this study was conducted to assess successive biological changes in proximal contact tightness PCT in class II direct composite restorations and the adjacent teeth by applying sectional matrix system along with different contact forming instruments.

**Methods:**

72 direct compound class II composite restorations were performed in patients aged 18–40 years and divided into 4 groups: Group I (n = 18): proximal contact was restored with Palodent plus sectional matrix system, Group II (n = 18): Trimax as contact forming instrument, Group III (n = 18): Perform as contact forming instrument and Group IV (n = 18): Contact pro as contact forming instrument. All contact forming instruments were used along with Palodent plus matrix system. PCT was measured using a digital force gauge before (T0), immediate post operative (T1) and at 3 (T2), 6 (T3), 9 (T4), and 12 months (T5) after restorative treatment. Using One-Way ANOVA, Tukey’s post hoc test, and Bonferroni correction, PCT values were compared between groups before and after the intervention restoration. Meanwhile, for comparisons within groups, a paired t-test was conducted (*p* ≤ 0.05).

**Results:**

Contact forming instruments combined with Palodent plus sectional matrix system achieved better PCT. Trimax led to a statistically considerable tighter proximal contacts than the other groups (p < 0.05). No statistically significant difference was found in PCT between Contact pro-2, Perform and Palodent plus sectional matrix system. By means of multivariate analysis, the PCT between both T0 and T1 were increased (p < 0.001) and then it decreased till T5.

**Conclusions:**

The use of transparent contact forming instruments achieved greater PCT compared to Palodent sectional matrix system alone that gradually decreased throughout 12 months and reached the PCT between the natural teeth. Using Trimax system provided the tightest proximal contacts. Additionally, digital force gauge was confirmed as an inclusive and accurate method to quantify PCT.

**Trial registration:**

ClinicalTrials.gov NCT05749640: 24/5/2022.

## Background

Appropriate proximal contacts are critical in proximal restorations. In direct class II composite resin restorations, both anatomical and tight proximal contacts can avoid food impaction, periodontal disease, tooth movement, and cavities [1]. The form, size, and position of the proximal contact regions are determined by the contours of the anatomic surface of the neighboring proximal surfaces and either they are on the mesial or distal aspects of the teeth [2].

To achieve proper proximal contact in direct composite resin restorations, the clinical procedure must compensate for the thickness of the matrix as well as the polymerization shrinkage of the composite resin. Both proximal contact tightness and proximal contours are two key factors that are related to the establishment of proximal surface [3, 4]. The PCT is regarded to be of a dynamic nature that could be influenced by the type and site of tooth, the patient’s position, the restorative techniques, and the masticatory forces. At different times in one day, PCT has shown to differ with the periodontal ligament’s fatigue and alterations in viscoelastic characteristics owing to the potential role of circadian rhythms [5].

For placement of the composite resin, varied measures have already been made to sustain the anatomical structure of proximal contact area. Special instruments were utilized to achieve the optimal proximal contour such as diverse matrix systems, separation rings and wedges [6]. There are many different types of matrix systems on the market specifically designed to be utilized with composite resin. Using both sectional matrix systems with separating rings has resulted in more PCT than circumferential matrix bands [7–12].

To ensure good embrasure anatomy, it is usually suggested that matrices should be burnished or held versus the adjacent tooth and therefore warrant that the contact can be cleaned effectively [13]. The matrix band displacement towards the adjacent tooth contact has evidence both for and versus enhancing PCT [14–16]. Thus, additional techniques have been created to aid the operator during light curing by offering new instruments in applying pressure on the contact region [13]. These new tools, known as Contact Forming Instruments, were created to be pushed or pulled in the direction of the contact to generate additional separating force via the matrix band, hold the composite in place during the light-curing phase, and supposed to produce a composite bridge that stabilizes the matrix when it encounters adjacent tooth [13, 17].

Contact forming instruments come in various types and designs, each with its own unique features and advantages. Traditional contact forming instruments are typically made of stainless steel or other dental-grade materials and may not have a transparent head [14]. While transparent contact formers have a transparent or semi-transparent head, often made of resin or polycarbonate. This transparency allows for the passage of light during the light-curing process, ensuring that the composite resin is properly cured and bonded to the tooth. Transparent contact formers provide excellent visibility during the restoration process [13].

According to the United States Public Health System (USPHS) guidelines, PCT can be determined when dental floss is used to evaluate contact tightness by recording the ease of dental floss passing interdentally [18–21]. Utilizing standardized metal blades or strips of shim stock with various thicknesses is a different way of determining the contact tightness. The thickness at which the interdental proximal area initially prevented passage was recorded [18]. While most previous studies have relied on the conventional and less precise approach of using dental floss, this research introduces a cutting-edge innovation by employing a digital force gauge to measure PCT accurately [16, 22, 23]. Unlike the subjective nature of dental floss assessment, the digital force gauge offers a quantitative and highly accurate measurement of PCT. This advancement not only enhances the precision of PCT evaluation but also introduces a new level of objectivity and reliability to the process. By quantifying the force required to pass through the proximal contact, this study provides a more robust and evidence-based approach to assessing the quality of dental restorations [16, 22, 23].

This one-year clinical study aimed to investigate the consecutive biological alterations in PCT between class II direct composite restorations and adjacent teeth after placement using Palodent sectional matrix system and with different transparent contact forming instruments.

## Methods

### Subjects, study design and setting

**Fig. 1:**
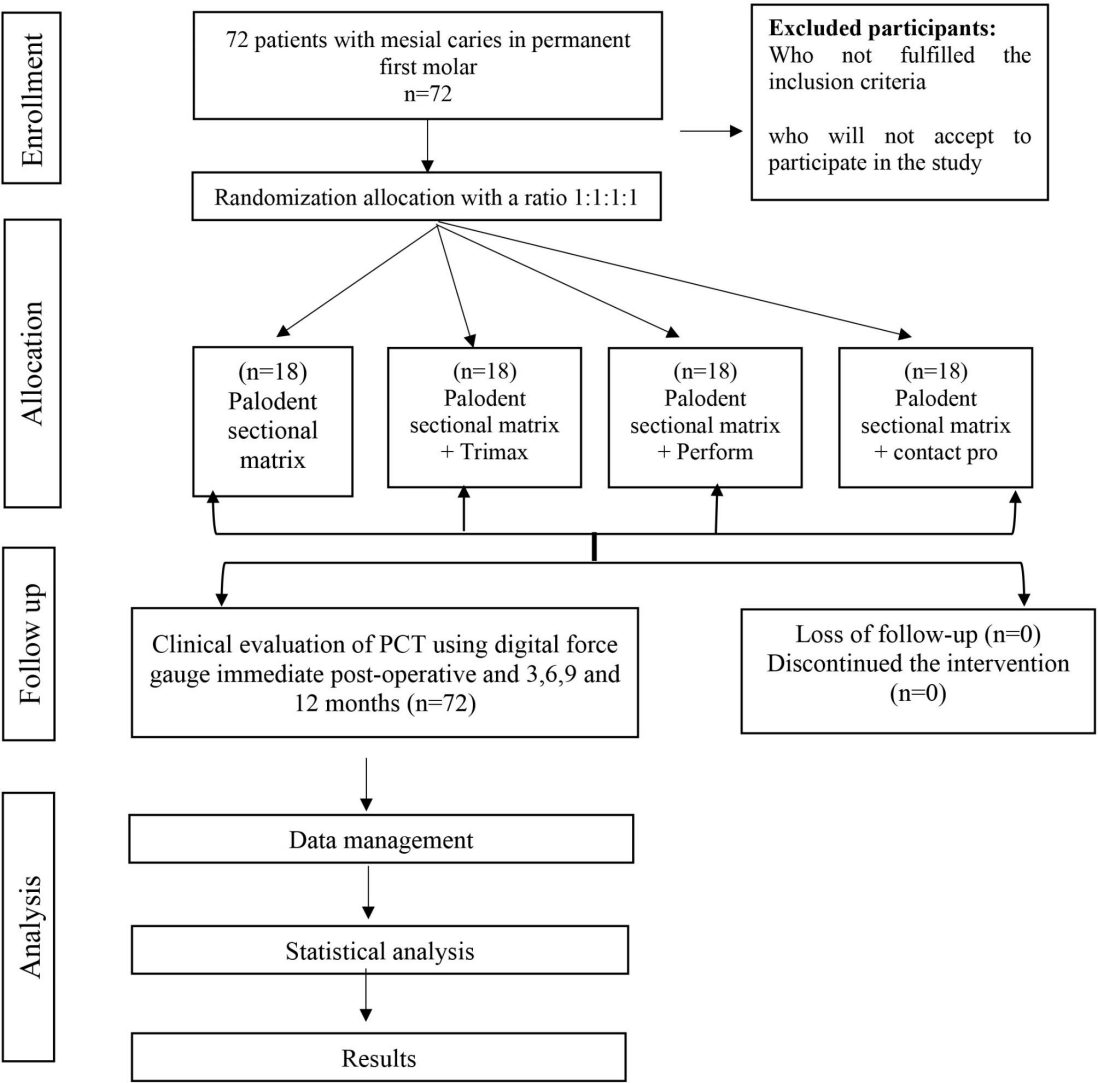
Consort chart of the clinical study

The current study was a controlled, randomized clinical trial that followed the Consolidated Standards of Reporting Trials (CONSORT) guidelines [24] (Fig. 1). Our current study was ethically approved by the Alexandria University Committee of Research Scientific Unit, with the reference number (0425-04/2022). The current study was also registered at ClinicalTrials.gov as NCT05749640. Participants were briefed on the protocols and signed a consent form before being included. Between May 2022 and May 2023, the clinical procedures and evaluations were performed.

### Inclusion and exclusion criteria

In the current study, 72 patients (18–40 years old) were included and treated in the conservative department clinics, Faculty of Dentistry, Alexandria University, at Alexandria, Egypt. Patients who met the following criteria were included [8, 23]: The presence of proximal caries on the mesial surface of the first molar in a digital x-ray with a score of 3 to 4 according to the radiographic International Caries Detection and Assessment System (ICDAS) [25], the antagonist, and the adjacent tooth making contact. The teeth should have no signs of pulpitis. Patients should also have inactive caries, no periodontal disease, and no major systemic disorders or allergies. Patients who were medically compromised or presented with traumatic malocclusion, cavitated mesial lesions and previously restored mesial surfaces were excluded from the current study.

### Sample size calculations

For power analysis, G*Power 3.1.9.2 software (Franz Faul, Universität Kiel, Germany) was used. The sample was generated using 80% power, 5% alpha error, and an effect size of 0.4338 based on previous research [23, 26]. A sample of 16 patients per group was needed, this was increased to 18 patients, yielding a total sample of 72 patients, to compensate for lost to follow up cases [27].

### Randomization and allocation concealment

Participants were randomly assigned with equal allocation to the four arms using a computer-generated list of random numbers in blocks of four. Allocation concealment was guaranteed by managing envelopes that are opaque, successively numbered and sealed till the date of candidate attendance. Once the patient consented to be included in the study, the envelope was opened, and each tooth was given the assigned treatment. The envelopes were prepared by a staff member not involved in any of the phases of the clinical trial.

### Intervention

**Fig. 2:**
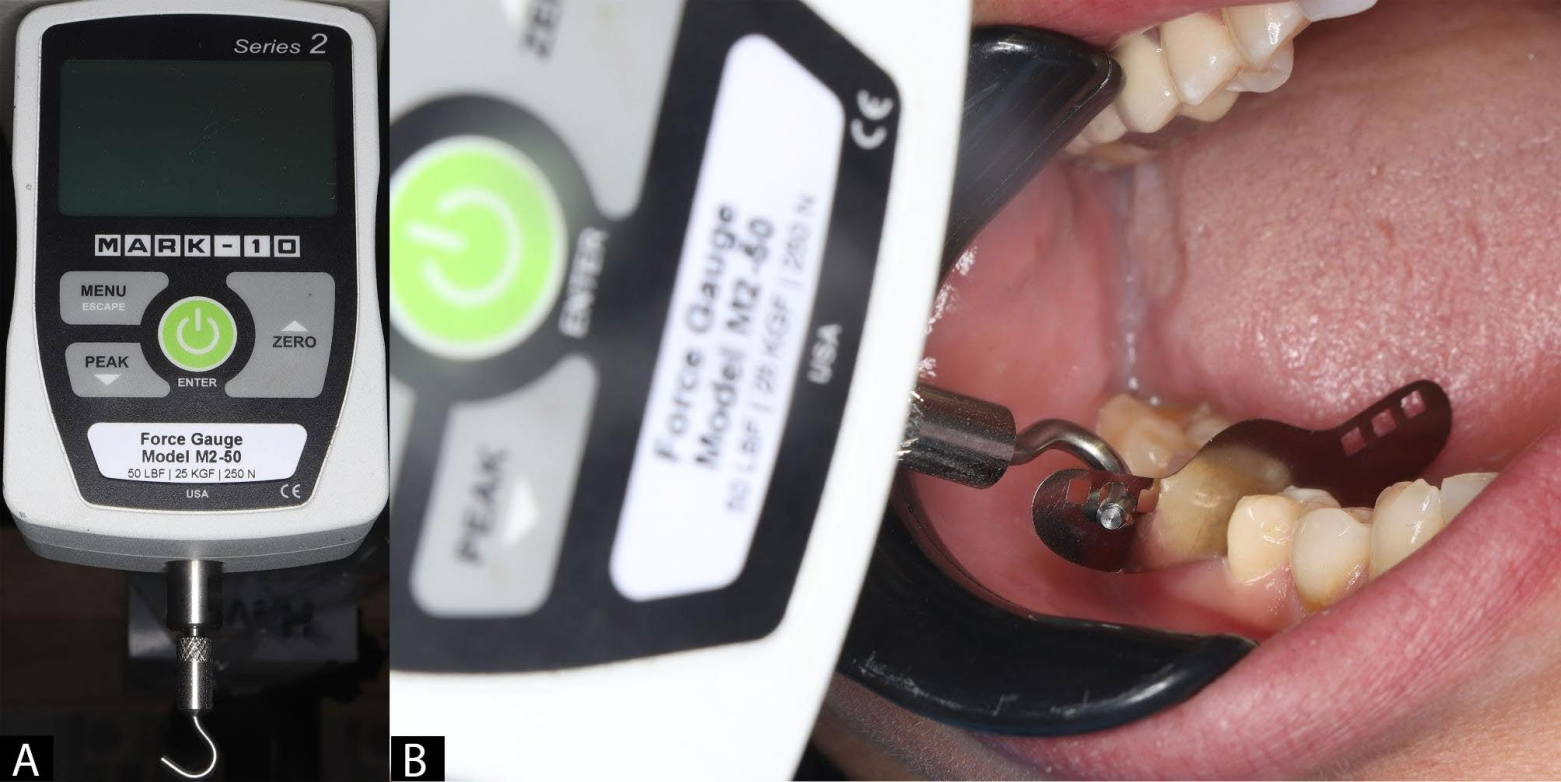
Showing **(A)** Mark-10 series 2 digital force gauge with small hook, **(B)** pulling of 0.05 ivory sectional matrix from buccal direction

In each group, PCT was measured using a digital force gauge (Mark-10 series 2, Mark Corporation, USA) that relied on a tooth pressure meter manufactured at Delft University of Technology in the Netherlands [2, 23]. The technique makes use of a 0.05 mm thick metallic strip (Perforated matrix band, TorVM, Moscow, Russia) that was introduced interdentally from an occlusal direction. The digital force gauge was linked to this metallic strip [2, 23] (Fig. 2).

**Fig. 3:**
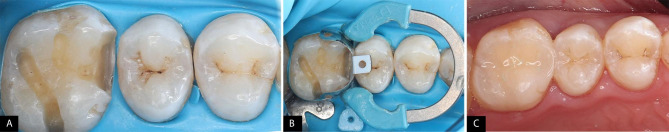
Showing **(A)** cavity preparation for direct class II composite restoration, **(B)** palodent plus sectional matrix system, **(C)** Final composite restoration

When the strip would progressively pull away in a buccal-lingual direction, the PCT was identified as the greatest frictional force. As the device was converted to peak-mode, the optimal for each measure, force was logged on the gauge’s screen. At each site, 3 measurements were made with optimum range of targets of 0.5 N. Measurement outcomes exceeding this range required repeating the measurement. Each single result of the measuring site was composed of these 3 results’ mean value. Then those mean values were recorded as (T0) baseline measurements [16, 17, 28]. Next to evaluating the PCT, class II cavity preparations were done in which the proximal contacts with the adjacent teeth were entirely cleared Fig. 3**(A)**, then sectional matrix system and contact forming instruments were applied following manufacturers` directions for use, and each tooth was restored with composite restoration according to the assigned groups.

In group I, both the pre-contoured sectional matrix EZ COAT and the separating ring (Palodent Plus Sectional Matrix System, DENTSPLY Sirona, USA) were applied, and the sectional matrix was stabilized by an appropriately sized wedge (Palodent Plus Wedge, DENTSPLY Sirona, USA) Fig. 3**(B)**. Selective enamel etching was performed using 37% phosphoric acid gel (3 M™ Scotchbond™ Universal Etchant Etching Gel, 3 M ESPE, St Paul, USA) for a duration of 15 s as recommended by the manufacturer. Excess acid was meticulously rinsed away to ensure complete removal of the etchant [12]. Following thorough drying and aided by a micro-brush, the adhesive (3 M ESPE Scotchbond Single Bond Universal Adhesive, 3 M ESPE, St Paul, USA) was applied to the prepared tooth and rubbed for 20 s, gently air dried for approximately 5 s to evaporate the solvent then light cured for 10 s in accordance with the manufacturer’s instructions. Polymerization was achieved using Elipar Deep Cure LED curing light (3 M ESPE, St Paul, USA) with an output irradiance of 1,470 mW/cm², designed for the polymerization of light-curing dental materials with a photo initiator for the wavelength range of 430–480 nm [8, 12].

For all restorations, the same composite resin (Filtek P60 Posterior Restorative, 3 M ESPE, St Paul, USA) was applied incrementally in 2 mm layers and light cured for 40 s as per the manufacturer’s guidelines. After the removal of matrix, restorations were post-cured for 40 s. Fine and ultra-fine diamond burs and the Sof-Lex system (3 M ESPE, St Paul, USA) was used to finish and polish the restorations as shown in Fig. 3**(C)** [8, 12].

**Fig. 4:**
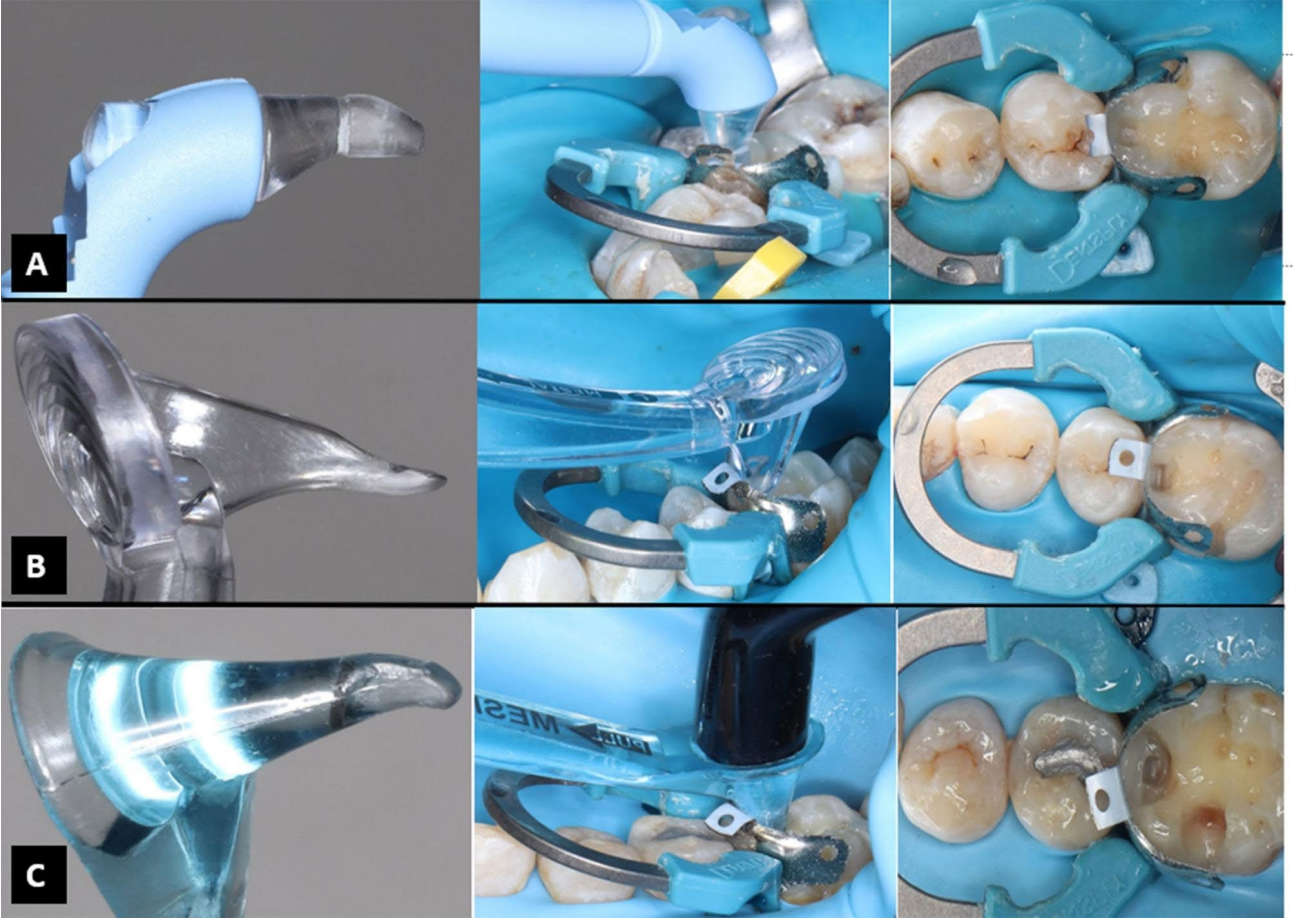
Showing contact forming instruments in action **(A)** Trimax in action, **(B)** Perform in action, **(C)** Contact pro 2 in action

In group II, the same method was followed, except a small layer of composite was applied to the prepared gingival step. During the light curing procedure, the handheld device (Trimax, AdDent, USA) was put within this uncured composite and pushed toward the direction of contact with the adjacent tooth until the composite and band were in close contact with the adjacent tooth Fig. 4**(A)**. In group III, the same procedure as previously described except for the handheld instrument (Perform, Garrison Dental Solutions, USA) was used Fig. 4**(B)**. In group IV, the same was done except the handheld instrument (Contact pro-2, CEJ, USA) was used Fig. 4**(C)**.

All the contact forming instruments employed were equipped with transparent heads that facilitated the effective application of light within the contact-forming instrument’s head during the light-curing process. Specifically, this transparency enables the curing of only the initial layer of composite resin as it passes through the head of the contact instruments during the pressing procedure [13]. All restorative treatments were performed by a single skilled operator (KA) in the conservative dentistry department, and PCT was recorded in all groups immediately after restorative treatment (T1).

### Calibration, blinding and follow-up examination

Before the study commencement, two outcome assessors (WM, AH) were trained and calibrated on the PCT measurement to ensure measurement reliability. Intra and inter examiner agreement was assessed using Intra Class Correlation Coefficient (ICC) that indicated a good agreement and it was ranged from 0.76 to 0.83. The two outcome assessors (WM, AH) were blinded concerning the previously performed interventions and the clinician (KA) only guided them to the treated tooth. PCT was measured between natural premolar teeth in the same quadrant and utilized as a control to capture changes in contact tightness. Differences in PCT were examined before (T0) and immediately after treatment (T1). All patients returned to the clinic and PCT was recorded at three-month (T2), six-month (T3), nine-month (T4), and one-year intervals after restorative treatment (T5).

### Statistical analysis

Data normality was assessed by means of Q-Q plots and the Shapiro Wilk test. Normal distribution was approved for all values; therefore, data were presented mainly using mean and standard deviation in addition to median, minimum, and maximum values. Repeated Measures of 2-Way ANOVA were made and then followed by pairwise comparisons with Bonferroni correction was performed to assess the matrix type effect, time, and the interaction between them on PCT. Using One-Way ANOVA, Tukey’s post hoc test, and Bonferroni correction, PCT values were compared between groups before and after the intervention. Meanwhile, for comparisons within groups, a paired t-test was conducted. Data were analyzed using IBM SPSS software package version 23 (IBM Corp., Armonk, NY, USA). The significance level was set at p value ≤ 0.05.

## Results

A sample consisted of 72 patients, accounting for total of 72 restorations; none of them reported contact sites inconveniences after 12 months with no dropout. No statistically significant difference was found between patients in regards of demographic distribution as shown in Table 1. Age was analyzed using One Way ANOVA and gender and arch are analyzed using Chi Square test.

**Table 1:** Demographic distribution of study participants

		Group I (n = 18)	Group II (n = 18)	Group III (n = 18)	Group IV (n = 18)	Test (p value)
Age: Mean (SD)		22.56 (1.25)	22.39 (1.10)	22.05 (1.21)	22.00 (1.19)	0.908 (0.442)
Gender	Males	10 (55.6%)	10 (55.6%)	12 (66.7%)	11 (61.1%)	0.635 (0.888)
	Females	8 (44.4%)	8 (44.4%)	6 (33.3%)	7 (38.9%)	
Arch	Upper	8 (44.4%)	7 (38.9%)	10 (55.6%)	8 (44.4%)	1.063 (0.786)
	Lower	10 (55.6%)	11 (61.1%)	8 (44.4%)	10 (55.6%)	

Both mean values and the standard deviation values of PCT at different follow up intervals among different matrix types are presented in Table 2. The usage of contact forming instruments combined with Palodent plus sectional matrix system gave rise to tighter PCT than using sectional matrix system alone. Usage of Trimax led to a statistically considerable tighter proximal contacts than the other groups (p < 0.05). No statistically significant difference was found in PCT between Contact pro-2, Perform and Palodent plus sectional matrix system as shown in Table 3

**Table 2:** Comparison of proximal contact tightness (PCT) at different follow up intervals among different matrix types

Follow up period	Palodent (n = 18)	Trimax (n = 18)	Perform (n = 18)	Contact Pro (n = 18)
	Mean ± SD			
**T0**	1.89 ± 0.33^ A,a^	2.14 ± 0.28^ A,a^	2.14 ± 0.13^ A,a^	1.92 ± 0.29^ A,a^
**T1**	4.83 ± 0.86^B,a^	5.88 ± 0.81^B,b^	5.31 ± 0.56^B,ab^	4.93 ± 0.63^B,a^
**T2**	4.73 ± 0.83^ C,a^	5.72 ± 0.64^ C,b^	5.04 ± 0.42^ C,ab^	4.74 ± 0.66^ C,a^
**T3**	4.42 ± 0.88^D,a^	5.35 ± 0.62^D,b^	4.65 ± 0.48^D,ab^	4.54 ± 0.62^D,a^
**T4**	4.30 ± 0.92^E,a^	5.14 ± 0.64^E,b^	4.43 ± 0.54^E,ab^	4.42 ± 0.59^E,ab^
**T5**	4.15 ± 0.82^ F,a^	4.94 ± 0.60^ F,b^	4.32 ± 0.58^ F,ab^	4.31 ± 0.57^ F,ab^

Different superscript lowercase letters denote statistically significant difference between study groups

Different superscript uppercase letters denote statistically significant difference between time points within each study group

**Table 3:** Pairwise comparison of PCT between different matrix types

Factors	Groups	Compared to	*P* value	Mean difference	95% CI	
					Upper limit	Lower limit
Matrix type	Palodent	Trimax	0.017*	-0.81	-1.51	-0.11
		Perform	1.00	-0.26	-0.96	0.44
		Contact Pro	1.00	-0.09	-0.79	0.61
	Trimax	Perform	0.218	0.55	-0.16	1.25
		Contact Pro	0.042*	0.72	0.02	1.42
	Perform	Contact Pro	1.00	0.17	-0.53	0.87

*Statistically significant at *p* value < 0.05

**Fig. 5:**
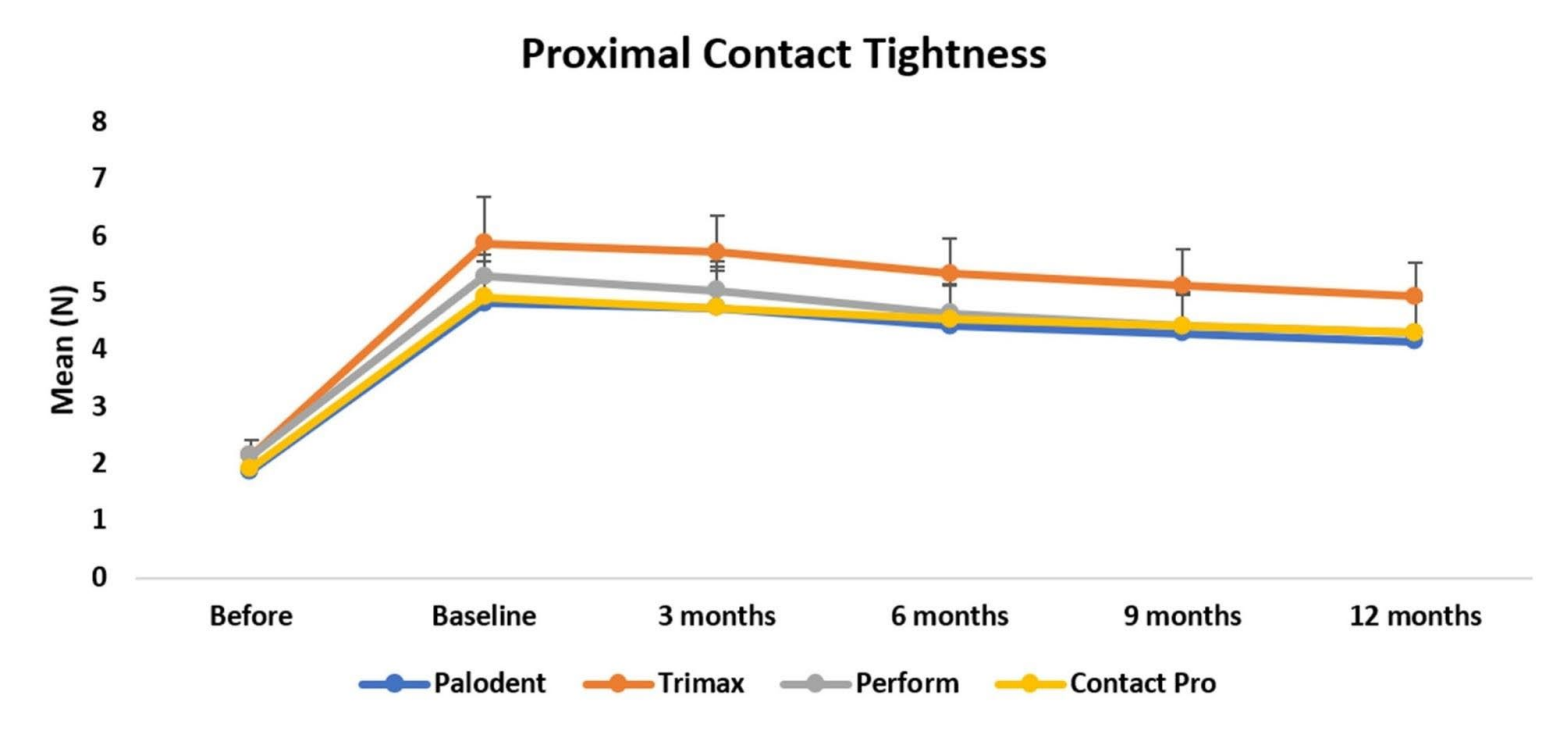
The PCT alterations between the restored teeth and neighboring teeth at T0, T1, T2, T3, T4 and T5

After restoration, a follow-up period of 12-month was performed and the PCT was considerably reduced and reached the PCT between the natural teeth. The PCT alterations between the restored teeth and adjacent teeth at T0, T1, T2, T3, T4 and T5 are shown in Fig. 5 which showed the gradual decrease in degree of tightness and the difference between the groups. PCT was also measured between natural premolar teeth (mesial control teeth) in the same quadrant where the restoration was performed and showed no statistically significant difference in PCT after 12-month follow-up period.

## Discussion

One of the most overlooked topics in posterior composite restorations is the interdental anatomy of a class II direct composite restoration. Promoting arch continuity and reducing impaction of foods must be the goals of the reconstructing of proximal contact. [29, 30]. Both PCT and proximal contours are two key factors that are related to the establishment of proximal surface [8, 31]. Precontoured sectional matrix is considered as an ideal tool for resembling the anatomy and emergence profile for restoring the proximal contour [6, 16, 32].

The international caries detection and assessment system “ICDAS” was employed to the current investigation as an evidence-based clinical scoring system [25, 33]. This common visual identification method serves as a guide for standardized caries detection. It was divided into six phases. It was developed to provide an accurate tool for the diagnosis and assessment of carious lesions. Additionally, it can be used for clinical practices, epidemiologists, and researchers [25]. A careful clinical examination can detect the presence of proximal caries. The examination should be achieved in a well-illuminated and clean field. Air dryness to each accessible surface is required. Also, cotton rolls under the tongue and in the vestibular space to enhance visualization and ensure dryness of teeth and gingiva. Furthermore, using a suitable instrument such as a mirror and probing with an explorer on the proximal is a must [20]. In the current study, caries lesions were detected by visual changes on the tooth surface, that all confirmed with digital x-ray [34].

In the present study, proximal contacts between the first permanent molar and second premolar were selected to be restored in all treated patients as they were found to have the tightest proximal contacts within a dental arch as concluded Dorfer et al. [2]. Moreover, all measurements were done at the same time at noon while patient in recline setting position and fasting mood to ensure standardization of all measurements as PCT has known to be dynamic physiological entity [8, 23].

Traditionally, to ensure proper proximal contact tightness, dental floss is used, and the proximal tightness is checked by permitting the passing of floss to a snap [19–21]. This technique was used in previous clinical studies and scored the contact tightness as ‟satisfactory”, ‟acceptable”, or ‟unacceptable”. But this assessment technique doesn’t have enough sensitivity for detecting any minor alterations in the strength of contact [16, 18, 23]. Thus, in this clinical study, digital force gauge and ivory matrix were used to measure accurately the PCT in newtons and easily detect the minor changes of PCT as previously described by Loomans et al [8, 23] who evaluated the PCT between posterior composite and adjacent teeth using this method, and also Ren et al [16] and Kandathilparambil et al [30] who investigated the biological alterations in PCT between the fixed implant and the neighboring teeth after placement via this method.

Sectional matrix systems with separating rings were shown to produce higher PCT than circumferential matrix bands as concluded by many studies [6–9, 15, 16] However, active coronal stabilization and separation with a separating ring can cause loss of contact and/or central and/or peripheral distortion. Both the central and peripheral distortion is frequently caused by rings that tent the matrix, open the gaps peripherally and press the contacting region against the adjacent tooth, resulting in it to dimple [6]. Thus, different transparent contact forming instruments were introduced aiming to produce more accurate proximal tightness [13, 17].

To the best of our knowledge, there is no available documented clinical data concerning evaluation of PCT using transparent contact forming instruments in combination with sectional matrix system. Therefore, this in vivo research was conducted to evaluate the PCT while using the sectional matrix system alone or in conjunction with different transparent contact forming instruments.

The study results revealed a tighter proximal contact with all matrices in comparison to baseline. There is a possibility that the tighter proximal contact may have been generated by a larger contact area produced by the matrices compared with the prior tooth structure that was indeed carious. Palodent Plus matrix system was utilized in all groups. This matrix system is designed to provide a standardized and anatomically shaped form for the restoration, which may contribute to creating a more consistent and well-shaped proximal surface compared to the carious tooth structure [31, 32]. Furthermore, the use of wedges in conjunction with these matrices aids in achieving a proper contact form. Previous studies have shown that Palodent Plus and Palodent matrix systems demonstrated minimal differences in contour compared to other matrix systems [32]. Moreover, in this study we found that teeth restored with a sectional matrix system in conjunction with a contact forming instrument had a greater PCT, this was similar in the idea to previous techniques which were raised like the use of prefabricated inserts and curing tips to achieve better contact [11, 15]. This can be explained by the effect of pressing the first layer of composite during the light curing procedure by the transparent hand instrument, which permit the blue light reach the pressed layer of composite and created a composite bridge that led to the matrix stabilization in contact with the adjacent tooth, ensuring intimate contact between the composite, matrix, and the adjacent tooth [13, 17].

Furthermore, trimax system showed significantly higher PCT than other tested contact forming instruments. This might be related to instrument geometry with its convex prongs that could simulate physiologic contacts in a more anatomical way. It was also presented as a full kit including many replaceable tips with different sizes that can accommodate in any cavity unlike other contact forming instruments that have fixed tips. This comes is in line with El-Badrawy et al. [11] who assessed the quality of proximal contacts of posterior composite restorations placed with 4 restorative techniques and concluded that using glass-ceramic inserts with convex geometry resulted in a better rate of acceptable proximal contacts in posterior composite restorations.

Moreover, the influence of a restorative intervention on the proximal contact was investigated during a period of 12 months. The PCT between Class II composite resin restorations and adjacent teeth was significantly decreased and generally reached the level of PCT between natural teeth. However, the proximal contacts at the natural premolar teeth showed no significant differences in tightness during the 1 year follow up period. Thus, it is likely that the observed differences in contact tightness at the treatment side are due to the restorative intervention. These findings are in accordance with Loomans et al. [23], Ren et al. [16] and Kandathilparambil et al. [30]. The observed decrease in PCT throughout follow up period in the current study could be explained by the proximal wearing of either the restorative material or the surface of the adjoining tooth. It was confirmed that after a period of 6 months, the mean proximal wear of a highly filled composite was ± 50 μm, whereas the enamel lost ± 5µ [35, 36]. Using identical composite resin for all restorations in this study has excluded it from being a research variable in the amount of proximal wears due to type of composite resin and/or application techniques. The other justification of this influence in PCT might be the ‘adaptation mechanism’ of the periodontal tissues. This ‘adaptation mechanism’ is based on the orthodontic principle of tooth movement as an additional tightness at the treatment site and spreads via both the proximal, mesial, and distal directions over more contact regions, leading to a newer balanced condition as concluded by Ren et al. [16].

In contrast to our findings, Prakki A et al. [19] and Sayed et al. [21] used dental floss to evaluate the proximal contact and reported no alterations in PCT throughout their follow up period. However, our different findings are probably owing to the more precise recording technique for PCT. According to the present research, no inconveniences were observed when the PCT was altered. No signs of food bolus impaction or periodontal inflammation were addressed during the follow up period indicating tight proximal contacts. Food impaction, tooth migration, periodontal problems, and eventually carious lesions have all been linked to a lack of or very loose proximal contacts in many studies [1, 32]. Additionally, acidic drink exposure [37] and occlusal contacts [38] can also have a significant influence on material hardness. These variables should be evaluated in future reports.

While this study has provided significant insights into PCT in direct Class II composite resin restorations, it’s crucial to recognize its limitations. The follow-up period was restricted to 12 months, and extending this duration would provide a more comprehensive understanding of how PCT may evolve over time. Moreover, this study concentrated on mesio-occlusal surface restoration for better standardization, but future investigations exploring different cavity design and matrix systems may provide further valuable insights. Additionally, future research should encompass an assessment of how these instruments influence not just PCT but also the precise location of proximal contacts, as this aspect was not fully addressed in our study. Despite these limitations, the study provides a valuable foundation for further research in this area, highlighting the importance of PCT in maintaining dental health. It is worth noting that throughout the course of the study, there were no instances of loss of follow-up or discontinuation of the intervention among the enrolled participants. This level of participant retention strengthens the study’s internal validity and the credibility of its findings. Future studies with larger cohorts and longer follow-up periods can build upon these findings to enhance our understanding of optimal restoration techniques and materials.

## Conclusions

The utilization of transparent contact forming devices achieved greater PCT than the Palodent sectional matrix system alone, which was gradually reduced over a 12-month period and reached the PCT between the natural teeth. The use of the Trimax system resulted in the tightest proximal contacts. Additionally, digital force gauge was confirmed as an inclusive and accurate method to quantify the PCT.

## Data Availability

The datasets used and analyzed during the current study are available from the corresponding author on reasonable request.
